# The effect of remuneration schedule on data completion and retention in the pregnancy eating attributes study (PEAS)

**DOI:** 10.1371/journal.pone.0251533

**Published:** 2021-05-13

**Authors:** Ndeah Terry, Leah M. Lipsky, Anna Maria Siega-Riz, Aiyi Liu, Tonja R. Nansel

**Affiliations:** 1 Division of Intramural Population Health Research, Social and Behavioral Sciences Branch, Eunice Kennedy Shriver National Institute of Child Health and Human Development, Bethesda, Maryland, United States of America; 2 Department of Nutrition and Biostatistics and Epidemiology, School of Public Health and Health Sciences, University of Massachusetts, Amherst, Massachusetts, United States of America; Arizona State University, UNITED STATES

## Abstract

Maximizing data completion and study retention is essential in population research. This study examined the effect of remuneration schedule and data collection modality on data completion and retention in the Pregnancy Eating Attributes Study cohort. Participants (n = 458) completed online surveys and attended six in-person study visits. Initially, remuneration was a prespecified amount per visit, then was changed mid-study to be prorated based on the number of forms completed. Additionally, survey data collection modality was changed to in-person at the sixth study visit. In this secondary data analysis, there was no effect of remuneration schedule on withdrawal rates or time-to-withdrawal. Survey completion was significantly lower under prorated remuneration at the first visit but did not significantly differ at subsequent visits. The lump sum group had significantly greater odds of completely the first and second trimester dietary record (OR = 4.1, OR = 2.6, respectively) then the prorated group but were almost half as likely to complete the dietary record at the 6-month postpartum visit (OR = 0.5). Survey completion at sixth visit was significantly higher for in-person versus online completion (68.6% vs. 93.1%). Findings suggest that remuneration schedule and data collection modality can impact completion of self- reported assessments.

## Introduction

Participants in research studies are typically provided with monetary remuneration as compensation for their time and effort. Remuneration may influence participation, data completion, or study retention [1–4], thereby impacting internal and external validity. Furthermore, recent advances in technology have facilitated the expansion of off-site, participant-initiated data collection, but whether this data collection modality impacts data completion or participant retention is unknown. Understanding how remuneration and data collection modality influence recruitment, retention, and data completion is critical for informing the most efficient and cost-effective design of human subjects’ research.

Participant remuneration is typically distributed via a predetermined schedule based on the time and effort associated with participation (e.g. assessment time, cost of transportation, child care, etc.), and evidence suggests that adequate remuneration may be critical to incentivize study participation [5–8] and retention [9]. Remuneration schedule, which refers to the system of dispersal of funds to participants throughout a study, varies across studies [10]. Remuneration may be provided conditionally (i.e. only after the completion of certain study tasks) in either a lump sum at a single time point or piecemeal, or as a prespecified amount paid unconditionally (i.e. not study task dependent) throughout the study according to milestones (i.e. number of visits completed) as specified by an institution’s human subjects’ review board. Another approach is to enter participants into a lottery for a gift card or monetary reward, if allowed by the institutional review board. To our knowledge, no studies have investigated how different payment schedules affect data completion and retention.

Additionally, studies use different strategies to collect data that may impact participant retention and data completion. Self-report measures may be collected, for example, at a central study location, at in-home assessments conducted by research staff, by telephone, or via participant self-administered online assessments. In-person survey completion at a central location may improve efficiency from the investigator’s perspective but necessitates physical space and on-site staffing and requires participant effort in terms of scheduling, transportation and parking. Alternatively, off-site participant-initiated survey completion via secure website or mobile applications may be more flexible and reduce participant burden associated with attending study visits at a central location. However, this approach may increase the cognitive burden associated with initiating and completing assessments, may be impacted by distraction, competing priorities, and limits participants to those who have internet or mobile access. Differences in these data collection modalities may influence participant retention and data completion. However, this has not been empirically investigated.

The purpose of this secondary analysis was to investigate the effect of remuneration schedule and data collection modality on participant data completion and retention in the Pregnancy Eating Attributes Study (PEAS). The study enrolled a cohort of women ≤12 weeks gestation to study eating behaviors and weight change from pregnancy through one-year postpartum. All participants were drawn from the same source population; however, two changes to study procedures were made based on findings from ongoing data collection monitoring. Because initial data completion rates during pregnancy assessments were lower than anticipated, the remuneration schedule was changed mid-study. Those recruited early in the study received a prespecified remuneration amount at each study visit regardless of how many self-initiated online forms they completed. Participants recruited later in the study were paid conditionally for each online form they completed. Additionally, in response to poorer completion of self-initiated, off-site online surveys during postpartum, data collection procedures were changed, and the number of required surveys were reduced for the final study visit such that participants completed surveys at the in-person assessment. These changes in study procedures facilitate an investigation into whether differences in remuneration schedule impacted data completion or withdrawal, and whether in-person versus off-site participant-initiated data collection modality influenced data completion.

## Methods

### Study design and participants

PEAS was a prospective observational study of 458 healthy pregnant women recruited at ≤12 weeks gestation and followed through one-year postpartum. Details of study recruitment and methods have been published elsewhere [11]. Participants were recruited from women receiving prenatal care at two obstetrics clinics in the University of North Carolina at Chapel Hill Healthcare System. Inclusion criteria were: confirmed pregnant ≤12 weeks gestation at enrollment; uncomplicated singleton pregnancy anticipated; age 18–45 years at screening; willingness to undergo study procedures and provide informed consent for her participation and assent for the baby’s participation; BMI ≥18.5 kg/m2; able to complete self-report assessments in English; access to internet with email; plan to deliver at the University of North Carolina Women’s Hospital; and plan to remain in the geographical vicinity of the clinical site for one year following delivery. Exclusion criteria included pre-existing diabetes; multiple pregnancy; participant-reported eating disorder; any chronic illnesses or use of medication that could affect diet or weight; psychosocial condition hindering participation in the study. Recruitment occurred from November 2014 to December 2016. Data collection was completed by August 2018. Protocols including modifications to the mode and remuneration were approved by the University of North Carolina at Chapel Hill institutional review board.

Anthropometrics and biospecimens were collected at in-person study visits once per pregnancy trimester and three times between delivery up to one year postpartum. Participants were also asked to complete self- administered online surveys on eating- and health-related behaviors and a 24-hour dietary recall outside of study visits via a secure study website. Participants logged on to the website with their username and password within specified time windows around each visit: window were from 6–12 weeks, 16–27 weeks, and 28–36 weeks gestation; and 4–14 weeks, 23–31 weeks, and 50–58 weeks postpartum. The website listed all required surveys for that window, with a link to the online survey form, and participants could complete them all at once or across multiple logins. When the visit window closed, the surveys were no longer accessible. Research assistants monitored the online data system to determine whether subjects completed the surveys and 24-hour dietary recalls. For subjects with incomplete forms, email reminders were given three weeks prior and phone reminders one week prior to window closure.

### Remuneration

Initially, participants received a prespecified remuneration at each study visit—$50 each for the first and third prenatal and first postpartum visit; $75 each for the second prenatal and second postpartum visit, and $100 for the final postpartum visit, for a total of $400 for completion of all visits. Due to low completion rates of online forms in the first several months of data collection, the remuneration schedule was changed in February 2016 for all subsequently recruited participants to be prorated based on the number of completed self-administered forms, under the hypothesis that a pro-rated remuneration schedule would increase survey completion. Under the prorated remuneration schedule, participants received $15 for each clinic visit, $15 for the dietary recall, and $3-$8 per online form (based on the form length), for a maximum total of $400. The first 284 participants received lump sum remuneration; the remaining 174 received prorated remuneration. Participants remained under the same remuneration schedule for the entire study.

### Data collection procedures at final study visit

When ongoing study monitoring indicated particularly low rates of data completion at the one- year postpartum visit, a second change in study procedures occurred. To ensure that the most important self-report measures were obtained at the final postpartum study visit, several surveys were eliminated from the assessment schedule, reducing completion time by about half, and participants were asked to complete all surveys during the clinic visit if they had not already completed them at home. This change occurred in March 2017, after 122 participants had already completed the final visit; 199 participants completed the final visit under the revised data collection modality.

### Statistical analysis

Differences between the two remuneration groups in socio-demographic characteristics were examined using Student *t*-tests for continuous variables and Pearson’s chi-squared test for categorical variables; education and income were included as covariates in all subsequent analyses by remuneration schedule. Remuneration schedule as a predictor of withdrawal by visit was modeled using Poisson regression. Group differences in study retention at one year postpartum were examined by Student *t*-test. Test for differences between remuneration groups in the percent of measures completed at each assessment period was determined by analysis of covariance. Differences in percent of participants who completed the 24-hour dietary recall at each visit by remuneration group were examined by logistic regression with prorated remuneration as the referent group. Data completion rates before and after the changes to the last postpartum visit were examined by Student *t*-test. All analyses were completed using SAS version 9.4.

## Results

Participants were mostly white, highly educated, and working at least part time (Table 1). There were no significant differences between remuneration groups in age, marital status, employment, education, race, ethnicity, and receipt of government aid. Differences in education and household income approached statistical significance and were, therefore, used as covariates in subsequent analyses.

**Table 1:** Baseline sociodemographics of women in PEAS^a^ under lump sum and prorated remuneration.

	Lump Sum^c^ (N = 2*8*4)	Prorated^c^ (N = 174)	
Demographics^*b*^	Mean ±SD or N(%)	Mean ±SD or N(%)	p^d^
**Age**	30.7±4.7	30.1±4.8	0.27
**Marital Status**			0.77
*Married*	217 (90.4)	116 (91.3)	
*Not Married*	23 (9.6)	11 (8.7)	
**Employment status**			0.32
*Full Time*	158 (65.8)	75 (59.0)	
*Part Time*	34 (14.2)	18 (14.2)	
*Not Working or Student*	48 (20.0)	34 (26.8)	
**Education**			0.07
*Less Than College*	73 (30.4)	31 (24.4)	
*College*	76 (31.7)	32 (25.2)	
*Graduate School*	91 (37.9)	64 (50.4)	
**Race**			0.23
*White*	188 (73.4)	98 (67.1)	
*Black*	42 (16.4)	25 (17.1)	
*Other or Mixed Race*	26 (10.2)	23 (15.8)	
**Ethnicity**			0.77
*Hispanic or Latino*	22 (8.6)	11 (7.7)	
*Not Hispanic or Latino*	219 (89.1)	130 (90.9)	
**Income-poverty ratio^e^**	3.71 ±1.2	4.08 ±0.2	0.08
**Household Size^f^**	3.0 ±1.2	2.9 ±1.2	0.67
**Any Aid Program^g^**			0.10
*No Aid*	204 (78.2)	124 (84.9)	
*Receives Aid*	57 (21.8)	22 (15.1)	

a Pregnancy Eating Attributes Study a prospective observational study of 458 healthy pregnant women recruited at ≤12 weeks gestation and followed through 1-year postpartum.

^b^ Demographic data missing for 91 participants for household size, income, marital status, and education, 63 participants for race, 51 for program aid, and 76 participants for ethnicity

c Investigators changed the remuneration schedule mid-study. Participants enrolled earlier in the study received a “lump sum” remuneration (n = 284) at each of the clinic visits were paid a set amount in full regardless of the amount of survey measures completed. Participants enrolled later in the study received prorated remuneration (n = 174) according to the number of measures completed.

d Student *t*-tests for continuous variables and Pearson’s chi-squared test for categorical variables. Statistical significance at p = 0.05.

eIncome-Poverty Ratio is an index the represents family income compared to the poverty threshold.

f Household size is the number of people in the household.

g Aid Programs included SNAP (Supplemental Nutrition Assistance Program), WIC (Women, Infants, and Children), free school lunch program, social security benefits, supplemental security income disability benefits.

Of 458 participants enrolled, 367 remained in the study through delivery and 321 through one-year postpartum for an overall study retention rate of 70%. Among the participants that withdrew, 91 (20%) withdrew prior to delivery and 46 (10%) withdrew during postpartum. Reasons for withdrawal included 48 no longer willing to participate; 29 experienced miscarriage, stillbirth, or death of baby; 17 moved away or changed medical provider; 37 were noncompliant with study visits; and 6 developed conditions resulting in ineligibility. There was no significant difference in the time to withdrawal between the two remuneration schedules, and no interaction of visit with remuneration schedule on number of withdrawals (95% confidence interval [CI] = -0.11,0.52; p = 0.19). There were no significant differences in the number of withdrawals between the two remuneration groups (27% in the lump sum remuneration, 33% in the prorated remuneration, χ2 = 2.13, p = 0.14).

Survey completion was significantly lower under prorated remuneration than lump sum remuneration at the first trimester visit (Table 2). Completion rates did not significantly differ between remuneration schedules at the subsequent study visits. Similarly, participants in the lump sum remuneration group were more likely to complete the 24-hour dietary recalls at the first two visits, but less likely to complete dietary recalls at 6 months postpartum. No differences were observed at subsequent visits (Table 3).

**Table 2:** Survey completion by remuneration schedule^a^.

		Lump Sum		Prorated	
Study Visit	n	%complete (mean ± SD)	n	%complete (mean ± SD)	p
Pregnancy					
1st Trimester	267	87.8±1.4	160	82.4±1.9	0.02
2nd Trimester	253	78.8±2.5	146	71.1±3.5	0.08
3rd Trimester	238	78.5±2.7	129	76.7±3.8	0.70
Postpartum					
4–6 Weeks	231	64.6±3.0	129	67.3±4.1	0.59
6 Months	221	60.7±2.9	126	67.0±3.9	0.19

a Analysis of covariance of percent of survey measures completed by participants in each remuneration group controlling for education and income. Values are mean ±SD. Statistical significance at p = 0.05.

**Table 3:** Dietary record completion (n, %) and OR (95% CI) of dietary record completion associated with remuneration schedule^a^.

		^b^ Lump Sum		^b^ Prorated		
Study Visit	n	(%) Complete	n	(%) Complete	Odds Ratio	95% CI
Pregnancy						
1st Trimester	267	82.8%	160	66.9%	4.1	2.0–8.5
2nd Trimester	253	73.9%	146	56.9%	2.4	1.3–4.2
3rd Trimester	238	69.3%	129	62.8%	1.4	0.8–2.4
Postpartum						
4–6 Weeks	231	58.0%	129	57.4%	1.1	0.7–1.8
6 Months	221	48.0%	126	61.1%	0.5	0.3–0.9

a Logistic regression on percent of participants completing diet records controlling for education and income with prorated remuneration as referent group.

b Investigators changed the remuneration schedule mid-study. Participants enrolled earlier in the study received a “lump sum” remuneration at each of the clinic visits were paid a set amount in full regardless of the amount of survey measures completed. Participants enrolled later in the study received prorated remuneration according to the number of measures completed.

Data completion at the one-year postpartum visit was significantly higher when participants completed surveys at the study visit, with a mean±se of 68.6±3.9% survey completion prior to procedural change, versus 93.1±1.8% afterward, (p < .001).

## Discussion

To increase data completion in this study of women assessed during pregnancy and postpartum, investigators changed the remuneration schedule approximately midway through data collection by linking remuneration amount to completion of each self-report measure rather than using a lump-sum remuneration schedule. Study findings indicate that the prorated remuneration schedule resulted in lower data completion than lump sum remuneration at initial study visits. No differences were observed in data collection at later study visits, and retention and time to withdrawal were unchanged. As such, the findings are contrary to the research team’s hypothesis and intention for changing the remuneration schedule mid-study. In contrast, changing data collection modality from off-site, participant-initiated to in-person assessment at the one- year postpartum visit had the most significant effect on data completion, with substantially higher data completion after the modality change.

The absence of an effect of remuneration schedule on retention suggests that other factors likely influenced withdrawals. Motivations for research participation previously reported include scientific interest or curiosity, the desire to further scientific knowledge, and humanitarian reasons [12]. Intrinsic motivators such as willingness to help medical research, improving the knowledge of science, and altruism are the most frequent reasons pregnant women report entering clinical studies [13]. Although payment is typically expected for study participation, participants in one study in a lower-income South African population reported they were willing to participate even if no compensation were provided [14]. Monetary compensation may be among the top motivating factors in populations of low-income or disproportional unemployment [15]. The PEAS sample was different in that on average women were highly educated and of relatively high income. While intrinsic motivators and monetary compensation may motivate research participation, retention may be impacted by unrelated issues such as changing family circumstances, health events, or job responsibilities [16, 17]. Multiple retention strategies that have been shown to increase retention rates [18–20] were used in PEAS including periodic newsletters, holiday cards, and provision of convenient times and locations for study visits. Therefore, retention in the PEAS sample may be more attributable to the various strategies used, rather than remuneration schedule.

While lower data completion under prorated remuneration was unexpected, results may be consistent with previous findings suggesting that monetary incentives may undermine intrinsic motivation [21, 22]. While task-noncontingent monetary rewards like lump sum remuneration have shown no impact on intrinsic motivation [23], task-contingent rewards like prorated remuneration have been found to decrease intrinsic motivation and reduce performance. As such, the prorated remuneration schedule used in PEAS could have decreased participants’ intrinsic motivation (i.e. a motivation shift from intrinsic to extrinsic), thus resulting in lower data completion rates. However, this explanation would not account for the lack of differences observed by remuneration schedule at later study visits. Additionally, the amount offered per survey under prorated remuneration may not have been adequate to motivate participants to complete surveys given the income levels of our participants. One study assessing performance quizzes and volunteer tasks at different payment levels suggests that effect of monetary incentives in small amounts can be detrimental to performance [24]. Thus, these findings taken together with previous work suggest that larger lump sum payments may lead to more favorable data completion and retention, and partitioning remuneration into a smaller series of payments may be a deterrent to data completion and retention.

Analysis of the one-year postpartum visit indicated that survey completion was dramatically improved by administering the surveys in-person during the study visit and reducing the number of surveys rather than relying on patient-initiated survey completion offsite. While logistical issues at the clinical site did not allow for in-person survey administration during pregnancy, and there is no literature directly comparing in-person survey administration versus off-site participant-initiated survey administration, these findings suggest that in-person administration of measures should be used whenever feasible and underscore the need to determine methods to improve self-administered survey completion.

Study findings should be interpreted in light of several limitations. Participants were not randomized into the remuneration groups; however, there were no significant differences in the socio-demographic characteristics between groups and no known historical changes across the study period (e.g. changes in study procedures, eligibility criteria, recruitment rates, or the population served by the clinics) that would impact comparability of the two groups. The study sample was largely well-educated with limited socioeconomic or racial diversity, and from a single geographic region; thus, findings may not be generalizable to participants with different demographic characteristics.

## Conclusion

Findings from this study indicate that remuneration schedule and data collection modality can impact completion of self-reported assessments. Changing the remuneration from a lump sum, task-noncontingent approach to a task-contingent prorated system resulted in lower data completion rates at initial visits but did not result in differential data completion at later visits. In contrast, changing the data collection modality from off-site, self-initiated to in-person resulted in substantial improvement in data completion. Further research is needed to understand how remuneration practices and data collection modality intersect with the economic status and demographics of diverse populations to influence data completion and retention.

## Supporting information

S1 Data (CSV)
